# Comparison of the Sustainability Effects of High-Intensity Interval Exercise and Moderate-Intensity Continuous Exercise on Cognitive Flexibility

**DOI:** 10.3390/ijerph18189631

**Published:** 2021-09-13

**Authors:** Shudong Tian, Hong Mou, Qun Fang, Xiaoxiao Zhang, Fanying Meng, Fanghui Qiu

**Affiliations:** 1 Department of Physical Education, Qingdao University, Qingdao 266071, China; tsdtyxy@163.com (S.T.); mouhong2021@126.com (H.M.); qfang_qdu@163.com (Q.F.); 2 School of Psychology, Shanghai University of Sport, Shanghai 200438, China; xxzhang07282021@163.com; 3 Institute of Physical Education, Huzhou University, Huzhou 313000, China; 02730@zjhu.edu.cn

**Keywords:** acute exercise, cognition, more-odd shifting, time course

## Abstract

This study examined the immediate and sustained effects of high-intensity interval exercise (HIIE) and moderate-intensity continuous exercise (MICE) on cognitive flexibility in young adults. Participants (*n* = 56) engaged in (1) a session of HIIE, involving 10 sets of one-minute treadmill running at an intensity targeting 90% heart rate reserve (HRR) interspersed with self-paced walking at 50% HRR; (2) a session of MICE, involving a 20 min treadmill running at an intensity of 40–59% HRR; and (3) a control session, involving 24 min of resting on separate days in a counterbalanced order. Using a more-odd shifting task, cognitive flexibility was assessed before the intervention (t_0_), immediately after the session (t_1_), and then at 30 min (t_2_) after the session. During the more-odd shifting task, the switch cost of response time (RT) immediately after the HIIE was significantly reduced compared to that before exercise, suggesting beneficial effects on cognitive flexibility. Additionally, the impacts of HIIE were maintained for 30 min post-exercise. However, improved cognitive flexibility was not observed until 30 min after the MICE intervention. HIIE might represent a time-efficient approach for enhancing cognitive flexibility.

## 1. Introduction

Cognitive flexibility represents an ability to shift perspectives or approaches between cognitive sets by altering behavior and actions according to changing conditions [1]. Such ability has been considered an important component of executive function [2]. Cognitive flexibility is predictive of social competence and plays an essential role in problem-solving [3]. Cognitive inflexibility refers to the tendency of people to focus on their own thoughts or behaviors, thus limiting their flexible problem-solving, inhibiting the switch from current thoughts and behaviors to another [4]. Previous studies have indicated that acute exercise produces transient positive effects on executive function [5,6,7,8,9]. However, to our knowledge, only a few studies examined the effects of acute exercise on brain health, using improvement in cognitive flexibility as a research endpoint [5]. Most of these studies used the moderate-intensity continuous exercise (MICE) as an intervention and found improved cognitive flexibility in both young adults [10] and older adults [11]. A recent meta-analysis showed greater improvements in executive function with acute MICE in comparison to acute low intensity or high intensity continuous exercise [7]. Acute high-intensity interval exercise (HIIE) is a more novel and time-efficient physical activity [12], which is now acknowledged as a key approach for cognitive and mental health [13] and is typically associated with temporary improvements (i.e., enhanced affect, release of endorphins, increased cerebral blood flow) [14]. HIIE elicits greater benefits on health-related fitness and cognitive function in comparison to MICE [15,16]. Further evidence has found a positive effect of HIIE on cognitive flexibility [17,18]. However, no research to date has made direct comparisons between HIIE and MICE regarding the effects on cognitive flexibility.

There is mounting evidence that the improved cognitive performance induced by acute exercise can be sustained for a certain period of time. Experimental studies have shown that benefits of acute exercise on executive function such as inhibitory control [19] and working memory [20] can persist up to 30 min post-exercise. Studies have proposed that the sustainability of changes in cognitive performance might depend on the protocol of the preceding exercise session. For example, Tsukamoto et al. [21] have demonstrated that HIIE and MICE protocols can improve Stroop task performance (a measure of inhibitory control) immediately after exercise. Whereas the enhanced performance in the Stroop task lasts for 30 min after the HIIE session, the improvement associated with the MICE returns to the pre-exercise levels. In addition, various studies have shown that intermittent exercise significantly improves performance in the flanker task (a measure of inhibition control) for over 60 min [22], but this improvement can be preserved up to 30 min after the continuous exercise [19]. However, it is unclear whether there is a difference in the effect of HIIE on the sustainability of improved cognitive flexibility compared to MICE.

Acute HIIE as a more time-efficient strategy has comparable or better results than acute MICE in terms of cognitive improvement [14]. HIIE has generated significant international interest in recent years and is the second highest training trend for 2020 [23]. Accordingly, the present study aimed to examine the immediate and sustained effects of acute HIIE and acute MICE on cognitive flexibility aspect of executive function in young adults. According to previous studies which demonstrated that different exercise intensities and modalities are potential moderators of exercise-induced executive control [6], we hypothesized that HIIE would elicit a more positive and sustained improvement in cognitive flexibility compared to MICE and control sessions.

## 2. Materials and Methods

### 2.1. Participants

Sample size was calculated via the statistical power calculation (G*power 3.1.9.2) on a medium effect size of 0.26 [24], using a 3 by 3 repeated measures design. Corresponding to the α level of 0.05 and a desired power (1-*β*) of 0.80 at the group level, a required sample size was 26 participants. To account for drop out we recruited fifty-six young adults (mean age = 20.18 ± 1.19 years) from the Qingdao University, China. Eligible participants should meet the following criteria: (1) right-hand dominant; (2) normal or corrected-to-normal vision and no color blindness; (3) free of any reported neurological or psychiatric diseases; (4) refrain from any moderate-to-vigorous physical exercise 24 h before the experiments; (5) refrain from stimulating drinks within 12 h of the study participation. The purpose of these inclusion criteria was to exclude the effect of moderators on the results of the experiment. All participants provided written informed consent after being informed of the potential risks. The research protocol and consents forms were approved by the Institutional Review Board of Qingdao University. Demographic characteristics and fitness data for all participants are provided in Table 1.

### 2.2. More-Odd Shifting Task

Cognitive flexibility was evaluated by the more-odd shifting task [25], which consisted of a series of digits from either 1 to 4 or 6 to 9. The task was generated by a computer program using E-Prime 2.0 (Psychology Software Tools Inc., Pittsburgh, PA, USA) and was displayed on a 15.6-inch monitor, the distance of which was 80 cm away from the participants.

All digits were presented for 2000 ms and separated by 1000 ms inter-stimulus intervals. The more-odd shifting task consisted of three types of blocks. Block A involves 16 non-switching trials in which the participants were asked to answer whether the number in black was greater or less than 5. Block B involves 16 non-switching trials in which the participants were asked to identify whether the number in green was odd or even. Block C consisted of 32 switching trials which requested the participants to determine the magnitude of the digit in black and the parity of the digit in green. Participants responded to each stimulus by pressing “F” or “J” button with their left or right index finger as quickly and accurately as possible. The task consisted of 2 switching blocks and 4 non-switching blocks in a counterbalanced order (i.e., ABCCBA). Response time (RT) and accuracy of the task were recorded. The mean RT from response-correct trials and accuracy were calculated for each trial type (switch and non-switch). In addition, we assessed the switch cost which was defined as the difference of RTs between the switching trials (i.e., block C) and the non-switching trials (i.e., block A and B) [26].

### 2.3. Exercise Protocols

**Figure 1:**
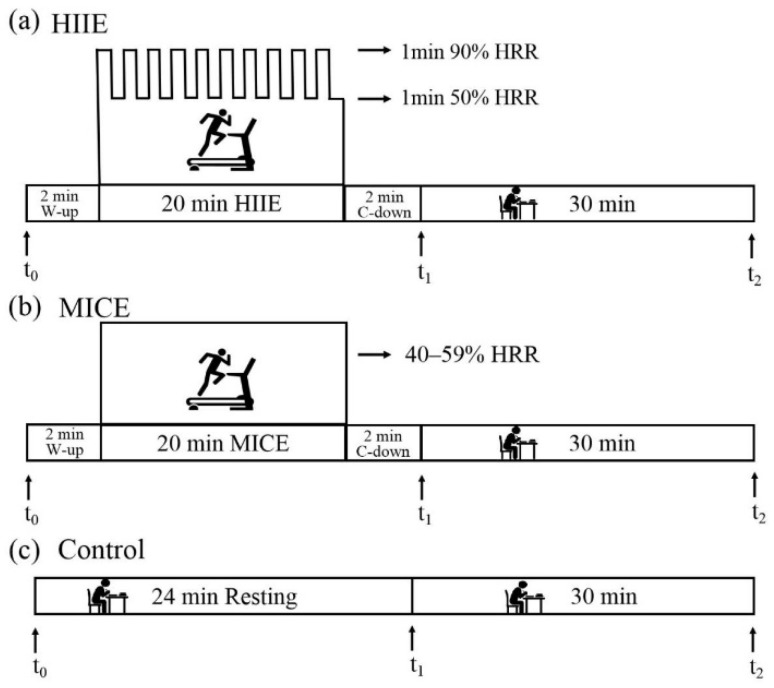
Time courses of HIIE (**a**), MICE (**b**) and control (**c**) sessions. Cognitive flexibility was assessed before the intervention (t_0_), and immediately (t_1_) and 30 min (t_2_) after the intervention. HIIE: high-intensity interval exercise. MICE: moderate-intensity continuous exercise. W-up: warm-up. C-down: cool-down.

Each participant was requested to attend three interventions of HIIE, MICE and control. Direct measurements of maximal heart rate (HRmax) is used by the graded exercise test (GXT) [27,28]. Heart rate reserve (HRR) was calculated based on HRmax to determine exercise intensity. The initial speed of the test was 8.5 km/h with a degree of 3%, then the treadmill speed was increased by 0.5 km/h every 1 min and the grade was kept constant until participants became volitional exhausted. Maximum volitional exhaustion was identified when the participant achieved at least two of the three following criteria: (a) a plateau in heart rate resulting in no longer rising with an increase in workload, (b) a peak HR ≥ age-predicted HRmax 208 − (0.7 × age) [29], and (c) Ratings of perceived exertion (RPE) ≥ 17. Resting heart rate (RHR) was obtained while seated using a Polar H10 heart rate strap (Polar, Kemple, Finland). Exercise intensity was appropriately tailored to each individual based on HRR. HRR corresponds to a defined percentage of the difference between HRmax and resting HR (HRR = HRmax − RHR) [30]. During the HIIE session, participants completed 10 bouts of repeated 1 min runs on a treadmill at an intensity targeting 90% HRR (90% HRR + RHR), interspersed with 1 min of self-paced walking at 50% HRR (50% HRR + RHR) [15,30]. During the MICE session, participants completed 20 min of running on a treadmill at an intensity of 40% to 59% HRR (40–59% HRR + RHR) [11,30]. Each exercise began with a 2 min warm-up and ended with a 2 min cool-down [15]. During the control session, participants sat quietly on a chair and read a book for 24 min [11]. Before each session, each participant was fitted with a Polar H10 heart rate strap, which was kept fitted until the end of each intervention. The protocols were illustrated in Figure 1.

### 2.4. Procedure

The study was conducted using a within-subjects, repeated-measures design. It included one HIIE session, one MICE session, and one control session conducted in a counterbalanced order. These three sessions were separated by at least one week and were completed at approximately the same time of the day. Before the experiment, participants completed the informed consent, the demographic sheet and the Physical Activity Readiness Questionnaire (PAR-Q) [31]. The purpose of PAR-Q was to exclude the potential risks. Exercise preparticipation health screening was performed for all participants to identify individuals who may be at increased risk of exercise-related sudden cardiac death and/or acute myocardial infarction [32]. Participants were instructed to practice 20 trials until 85% accuracy for the trial block was achieved before each experiment. In addition, all participants were asked to complete the shifting task before the intervention (t_0_) and at two time points after the intervention, including immediately (t_1_), 30 min (t_2_). Ratings of perceived exertion (RPE) [33] were assessed at 5 min intervals during HIIE and MICE interventions and average RPE was computed. According to the American College of Sports Medicine (ACSM) guidelines state that the RPE of high- and moderate-intensity exercise should fall within 14–17 and 12–13 RPE, respectively [34]. The purpose of measuring the RPE score is to compare it with the RPE score of the ACSM guidelines for exact exercise intensity. Participants received 150 RMB at the end of the entire session, and were told the detailed purpose and expectations of this research.

### 2.5. Statistical Analysis

For the RT analysis, incorrect trials were first removed and then an outlier correction was performed by separately excluding trials with an RT of 3 standard deviations from the mean for each task condition (switch and non-switch). Response accuracy and RTs were analyzed using a 3 (session: HIIE, MICE, and control) × 3 (time point: t_0_, t_1_ and t_2_) × 2 (task condition: switch and non-switch) three-way repeated-measures analysis of variance (RM ANOVA). Switch cost was examined separately for RT (switch − non-switch) [17] using a 3 (session: HIIE, MICE, and control) × 3 (time point: t_0_, t_1_ and t_2_) RM ANOVA. Mauchly’s test was used to examine spherical data, and the Greenhouse–Geisser correction was used to analyze non-spherical data. The Shapiro–Wilk normality test was applied to confirm normal distribution of data before the ANOVA. Paired-samples *t*-test with Bonferroni adjustments for multiple comparisons were applied for post hoc analysis [11]. The *p*-value of 0.05 was selected as the cutoff point for statistical significance. Effect sizes were presented by partial squared (*η*^2^) values as measures for main and interaction effects. All statistical analyses were performed by the Statistical Package for Social Sciences software (SPSS version 25.0, Chicago, IL, USA).

## 3. Results

### 3.1. Response Time

**Figure 2:**
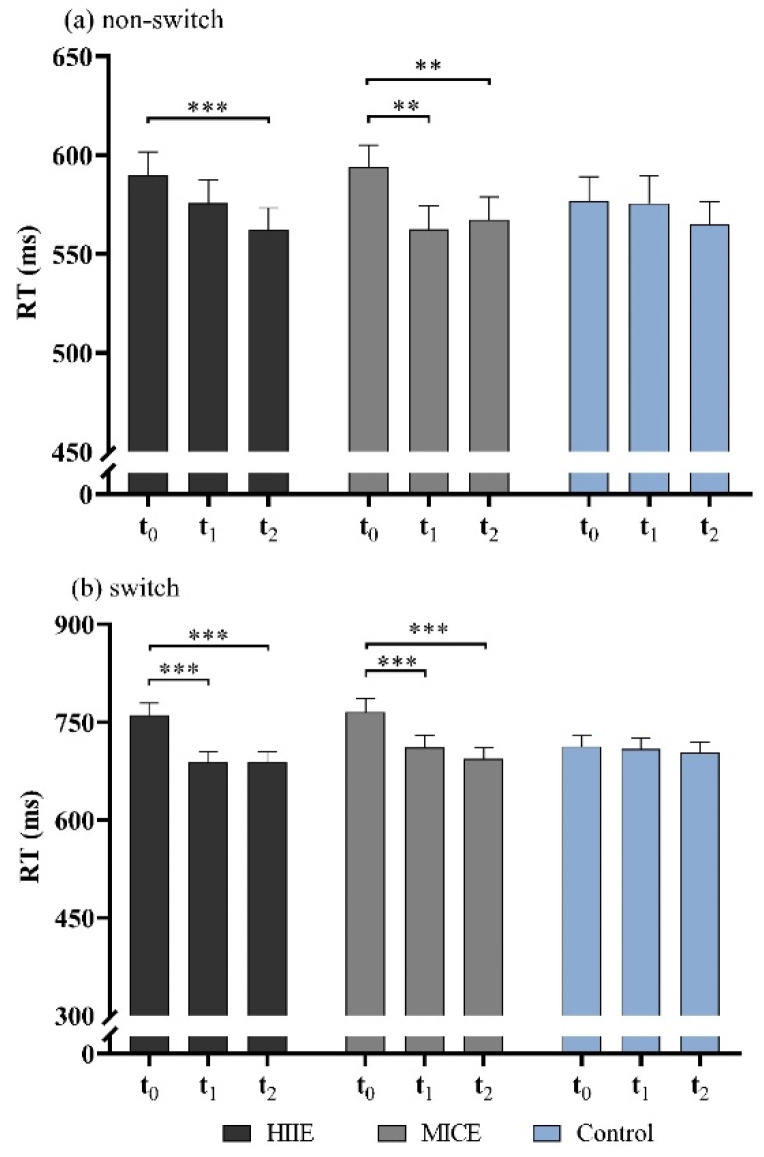
RTs for non-switching condition (**a**) and switching condition (**b**) in the more-odd shifting task. HIIE: high-intensity interval exercise; MICE: moderate-intensity continuous exercise. (** *p* < 0.01, *** *p* < 0.001).

The there-way RM ANOVA revealed a significant interaction for time point by task condition (*F*_(2,56)_ = 11.96, *p* < 0.001, *η*^2^ = 0.42), and time point by session (*F*_(4,54)_ = 9.71, *p* < 0.001, *η*^2^ = 0.42). In addition, there was a significant session × time point × task condition interaction effect (*F*_(4,54)_ = 3.16, *p* = 0.021, *η*^2^ = 0.20). Comparisons in the interactions between session, time point, and task condition revealed that RT in the non-switching condition was significantly slower before the MICE intervention compared to immediately (*p* = 0.001) and 30 min (*p* = 0.006) after the MICE intervention. For the HIIE intervention, RT in non-switching condition was significantly slower before the intervention compared to 30 min after intervention (*p* < 0.001). RT for the switching condition immediately after HIIE was significantly shortened compared to that before HIIE, and this shortened RT was sustained during the 30 min post-exercise recovery (*p* < 0.001 for all). Similar results were found after MICE, with a decreased RT immediately after MICE, and this improvement lasted for up to 30 min after exercise (*p* < 0.001 for all). There was a significant main effect for time point (*F*_(2,54)_ = 32.40, *p* < 0.001, *η*^2^ = 0.55). The post hoc test showed that RT was significantly slower before the intervention (666.84 ± 111.81 ms) compared with immediately (637.26 ± 109.63 ms, *p* < 0.001) and 30 min (630.10 ± 103.96 ms, *p* < 0.001) after the intervention. A significant main effect of task condition was identified (*F*_(1,57)_ = 114.85, *p* < 0.001, *η*^2^ = 0.67), with longer RTs in the switching condition (715.10 ± 127.44 ms) compared with the non-switching condition (574.37 ± 89.49 ms). No significant differences were detected among time points for switching and non-switching trials in the control session (*p* > 0.45 for all). No main effect for session (*F*_(2,54)_ = 0.25, *p* = 0.78, *η*^2^ = 0.009) or interaction of session by task condition (*F*_(2,54)_ = 0.54, *p* = 0.59, *η*^2^ = 0.02) was observed (Figure 2a,b).

### 3.2. Switch Cost

**Figure 3:**
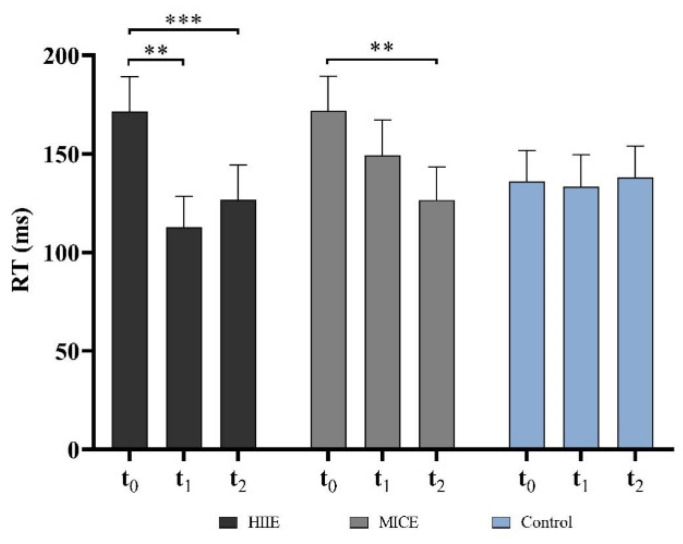
Switch cost of the more-odd shifting task. (** *p* < 0.01, *** *p* < 0.001).

The two-way RM ANOVA revealed a significant effect for interaction of session by time point (*F*_(4,54)_ = 3.16, *p* = 0.021, *η*^2^ = 0.20). The interaction contrasts revealed that switch cost was significantly lower immediately (*p* = 0.001) and 30 min (*p* = 0.001) after the HIIE intervention compared to that before the HIIE intervention and was significantly lower 30 min (*p* < 0.001) after the MICE intervention compared to before the MICE intervention. There was a significant main effect for time point (*F*_(2,56)_ = 11.96, *p* < 0.001, *η*^2^ = 0.42). The post hoc test showed that the switch cost was significantly higher before the intervention compared to immediately (*p* = 0.001) and 30 min (*p* < 0.001) after the intervention. No significant difference was found in the switch cost between the time points in the control session (*p* > 0.9 for all) (Figure 3).

### 3.3. Accuracy

**Figure 4:**
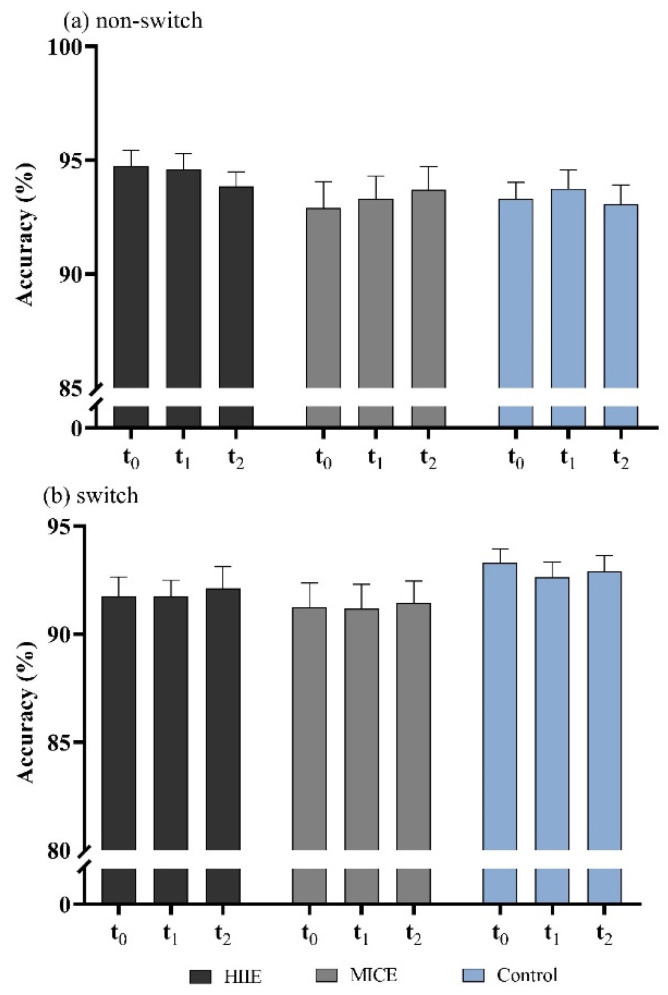
Accuracy for non-switching trials (**a**) and switching trials (**b**) in the more-odd shifting task.

The there-way ANOVA revealed a significant main effect on task condition (*F*_(1,55)_ = 6.88, *p* = 0.013, *η*^2^ = 0.16), with greater mean accuracy for the non-switching (93.69 ± 6.29%) condition than the switching condition (92.04 ± 6.64%). There was a significant interaction between session and task condition (*F*_(1,55)_ = 6.88, *p* = 0.013, *η*^2^ = 0.16). No significant result was identified in the main effects of session (*F*_(2,54)_ = 0.51, *p* = 0.60) or time point (*F*_(2,54)_ = 0.001, *p* = 0.99) or interaction effects of time point × task condition (*F*_(2,54)_ = 0.45, *p* = 0.64), session × time point (*F*_(4,52)_ = 0.21, *p* = 0.93) or session × time point × task condition (*F*_(4,52)_ = 0.44, *p* = 0.78) (Figure 4a,b).

## 4. Discussion

This study evaluated the sustained effects of acute treadmill-based HIIE and MICE on cognitive flexibility in young adults. Participants performed better in a more-odd shifting task, with the improved performance lasting for at least 30 min after the intervention. The improvement in cognitive flexibility was only detected 30 min after the end of the MICE session. These findings suggest that both HIIE and MICE can improve cognitive flexibility. HIIE may be a more effective strategy than MICE in improving cognitive flexibility.

Exercise as a stressor can promote physiological and psychological arousal [21,35], and increase oxygen and blood flow to the brain [36], which can optimize the allocation of cognitive resources and promote the efficiency of cognitive processing [37]. This study showed that participants performed better in the cognitive flexibility task after the HIIE intervention, compared to the resting session. The findings are consistent with previous studies [17,18]. Using a modification of the number-letter task, Berse et al. [17] showed that the switch cost of RT generally declined and that the accuracy of the switch and non-switch significantly increased after HIIE, compared with the non-significant changes in the resting condition. However, comparable facilitation in cognitive flexibility was not observed in the cognitive tests immediately following MICE intervention, which was also substantiated by previous studies. For example, Schwarck et al. [38] found that no acute effects of MICE on cognitive flexibility were observed in young adults. In contrast, Chen et al. [11] tested the effects of MICE on switching task performance and found that 20 min of MICE was effective in improving cognitive flexibility in older adults. The inconsistencies related to the effects of acute exercise on cognitive flexibility may be attributed to the characteristics of participants, types of task paradigms assessed, duration of exercise intervention, and the time at which the cognitive task was administered [6,11]. Collectively, our findings suggest that in young adults, acute effects on cognitive flexibility are favorable to HIIE over MICE.

The present study demonstrated that the improved cognitive flexibility identified immediately after HIIE was sustained for at least 30 min. This result is comparable to the previous research which has examined other aspects of executive function including inhibition control [21,39] and working memory [20]. While sharing some similarities, the subdomains of executive control do have distinguishing characteristics. Martínez-Díaz et al. [20] reported similar improvements in the work memory assessed by the digit spans test and the improvement in working memory was maintained for 30 min after the HIIE session. Cooper et al. [39] examined high-intensity intermittent games-based activity on inhibitory control using a Stroop task and indicated that the benefits of HIIE can last up to 45 min after exercise. Collectively, not only does HIIE have a sustained effect on inhibitory control and working memory, but also has a delayed effect on cognitive flexibility. Cognitive flexibility performance was improved 30 min after the MICE intervention, rather than immediately, as indicated by lower switch cost. This result is consistent with the conclusions of a meta-analysis, which reported that effects of moderate intensity exercise on executive function were more pronounced after a delay exercise than immediately following the exercise [6]. Furthermore, Kamijo et al. [40] conducted a 20 min MICE intervention and found that cognitive performance improved within 30 min after the intervention. Decreased intraindividual variability 30 min after MICE relative to before exercise may have contributed to this result [40]. Intraindividual variability is regarded as reflecting the ability to monitor and maintain consistency in task performance during acute cognitive effort [41]. Thus, the decreased intraindividual variability 30 min after MICE is likely to be indicative of upregulation of cognitive flexibility to maintain task performance over the entire course of the shifting task.

In addition, our findings reflect that the positive effect of HIIE on cognitive flexibility is disproportionately larger in task conditions with greater cognitive demands (switch trials). Evidence has shown that selective cognitive improvements occur following acute exercise, with more significant benefits occurring for tests that require greater cognitive demands [16]. Several recent studies have confirmed that acute exercise has a disproportionately larger effect on performance of tasks involving higher executive function demands, including inhibitory control tasks [16,42] and working memory tasks [43]. Using a flanker task, Kao et al. [16] found that HIIE increased response accuracy selectively for incongruent trials. As for the RT, Cooper et al. [39] revealed that higher complex levels of Stroop task was improved after the HIIE intervention. Our results confirm previous findings that conditions with larger cognitive demands improve more significantly after HIIE.

Acute exercise may improve task performance through different mechanisms. It is understood that exercise induces an increase in brain-derived neurotrophic factor (BDNF) as a possible mechanism leading to improved cognitive flexibility [18]. BDNF is an activity-dependent protein that may also influence functional connectivity by increasing synaptogenesis and dendritic spine density, thus improving cognitive performance by increasing synaptic plasticity [44]. Robust expression of BDNF in the hippocampus is crucial in mediating the enhancement of exercise-induced cognitive flexibility [18]. Saucedo Marquez et al. [45] revealed that the HIIE protocol (10 × 1 min bouts at 90% of maximal HR, alternating with 1 min rest for a total duration of 20 min) is a more effective intervention for elevating BDNF levels than MICE. Higher BDNF levels may account for the immediately improved cognitive flexibility performance after HIIE intervention compared to MICE. Furthermore, increased levels of cerebral cortex activation may be another factor for the cognitive boosting effect of exercise. Previous studies have suggested that improved executive function (including cognitive flexibility) after acute exercise was associated with increased activity in left-dorsolateral prefrontal cortex and parietal regions of the brain [46,47], including increasing oxygen-rich blood flow to the brain [48] and enhancing psychological arousal level [47]. Tsukamoto et al. [21] demonstrated that Felt Arousal Scale [49] derived arousal level was significantly higher for HIIE than MICE immediately after the exercise. Therefore, HIIE might more efficiently modulate the central nervous system activation and better improve cognitive performance relative to MICE.

The current study has a few limitations that need to be taken into account in interpreting the findings. First, our study design could have resulted in practice effects due to the number of testing sessions. However, we counterbalanced the sequence of sessions and extended the interval between sessions to at least 7 days to minimize such effect by spreading any remaining practice effects across all conditions. Second, the 20-min duration and intensity of exercise selected for this study may be appropriate for young adults, but may need to be adjusted in people of other age levels or fitness levels. Third, this study compared the acute effects of HIIE and MICE on cognitive flexibility for only 60 min. In future studies we will investigate the effects with chronic or acute HIIE on cognitive flexibility over a longer period of time at least for few days.

## 5. Conclusions

The present investigation indicated the sustained effect of treadmill-based acute HIIE and MICE on cognitive flexibility in young adults. Specifically, the MICE intervention significantly facilitated cognitive flexibility within 30 min after exercise. However, the enhanced cognitive flexibility by the HIIE intervention was not only reflected immediately after exercise, but also lasted for 30 min after exercise. Thus, HIIE represents a time-efficient approach for enhancing cognitive performance. It is recommended to consider HIIE an effective approach to improve performance that relies on cognitive flexibility. By using HIIE to separate a long-hour task into a few intervals, people may achieve higher efficiency in the workplace or at school. Therefore, the current study implies practical values that should raise awareness of the public.

## Figures and Tables

**Table 1:** Demographic characteristics and fitness data (*M ± SD*).

Measures	
Anthropometric variables	
Sample size (*n*)	*n* = 56
Gender (male/female)	31/25
Age (years)	20.18 ± 1.19
Height (cm)	172.99 ± 9.07
Weight (kg)	65.68 ±13.51
BMI (kg/m^2^)	21.77 ± 3.26
Health measures	
HRmax (bpm)	192.65 ± 8.08
HRR (bpm)	131.20 ± 10.07
Mean HIIE HR (bpm)	164.67 ± 10.99
Mean MICE HR (bpm)	136.38 ± 5.07
HIIE RPE	16.03 ± 1.51
MICE RPE	13.00 ± 1.69

Note: BMI = body mass index; RPE = ratings of perceived exertion; HR= heart rate; HRR = heart rate reserve.

## Data Availability

The data presented in this study are available on request from the corresponding author.
